# Thoraco-lumbar Burst Fractures among Patients Admitted to Spine Unit of the Department of Orthopedics of a Tertiary Care Centre: A Descriptive Cross-sectional Study

**DOI:** 10.31729/jnma.8070

**Published:** 2023-03-31

**Authors:** Samaj Gautam, Krishna Prasad Paudel, Sunil Panta

**Affiliations:** 1 Department of Orthopedics, Bharatpur Hospital, Bharatpur, Chitwan, Nepal

**Keywords:** *fracture*, *injuries*, *prevalence*, *spine*

## Abstract

**Introduction:**

Burst fractures are the most common fractures in the thoracolumbar junction. Unstable burst fractures are mostly associated with neural injury. Early neurological and mechanical stabilisation are the goals of treatment. The aim of this study was to find out the prevalence of thoracolumbar burst fractures among patients admitted to the spine unit of the Department of Orthopedics of a tertiary care centre.

**Methods:**

This descriptive cross-sectional study was done in a tertiary care centre from 1 January 2021 to 31 December 2021 after receiving ethical approval from the Institutional Review Committee (Reference number: 079/80-11/BHG). Demographic details, mode of injury, morphology, neurological level, and neurological grade using the American Association of Spinal injury, Visual analogue Scale, Oswestry Disability Index and kyphotic angle were recorded. A convenience sampling method was used. Point estimate and 90% Confidence Interval were calculated.

**Results:**

Among 85 patients, the thoracolumbar burst fractures was found in 30 (35.25%) (26.73-43.77, 90% Confidence Interval). The mean age of patients was 39.73±13.91 years.

**Conclusions:**

The prevalence of thoracolumbar burst fracture was similar to other studies done in similar settings.

## INTRODUCTION

The transition from a kyphotic and stiff thoracic spine to a lordotic and mobile lumbar spine makes the thoracolumbar region (T_10_-L_2_) more vulnerable to fractures. Burst fractures are the most common fractures in this region and usually, these fractures are associated with neurological dysfunction.^1^ The aim of treatment of these fractures is to correct deformity, decompress the neural elements, stabilization and early rehabilitation. Surgical approaches can be anterior, posterior or both with comparable outcomes.^2,3^ Due to the less extensile and less morbid approach posterior transpedicular fixation has been the choice for stabilizing these fractures.^4^ Long segment posterior fixation (LSPF) involves a pedicle screw two levels above and two levels below the fracture.^5^

With an increasing number of cases and changes in our standard care of practice, more patients are treated surgically. There are minimal studies regarding thoracolumbar burst fractures managed with long-segment posterior fixation in our region.

This study aimed to find out the prevalence of thoracolumbar burst fractures among patients admitted to the spine unit of the Department of Orthopedics of a tertiary care centre.

## METHODS

This descriptive cross-sectional study was done from 1 January 2021 to 31 December 2021 at Bharatpur Hospital. Ethical approval was taken from Institutional Review Committee (Reference number: 079/80-11/BHG). All the admitted patients within the spine unit of the Department of Orthopedics with spinal injuries during the study period were included in the study. The patients with associated head or pathological fractures and patients with American Association of Spinal Injury (ASIA) neurological status Grade A were excluded from the study. A convenience sampling method was done. The sample size was calculated using the following formula:


$$\begin{array}{ll} \text{n} & ={\text{Z}}^{2}\times \frac{\text{p}\times \text{q}}{{\text{e}}^{\text{2}}}\\ & =1.645^{2}\times \frac{0.50\times 0.50}{0.10^{2}}\\ & =68 \end{array}$$

Where,

n = minimum required sample size
Z = 1.96 at 95% Confidence Interval (CI)
p = prevalence taken as 50% for maximum sample size calculation
q = 1-p
e = margin of error, 5%

The proforma was filled with a demographic profile of the patient, mode of injury, level of injury, neurology according to the ASIA scale, surgery time, the interval from admission to surgery time, duration of hospital stay and complications of surgery if any recorded.

Clinical and neurological examination was done for neurological deficits as per the ASIA scale.^6^ Baseline X-rays, computed tomography (CT Scan) and magnetic resonance imaging (MRI) were done. The kyphotic deformity was measured by Cobb's method. Cobb's Angle is the angle formed between the line parallel to the superior end plate of upper vertebrae above fractured vertebrae and the line parallel to the inferior end plate of lower vertebrae below fracture vertebrae. The preoperative and the immediate postoperative kyphotic angles were noted. Radiological (Cobb's angle), neurological (ASIA) and, functional parameters with 100 points for visual analog scale (VAS) and Oswestry disability index (ODI),^7,8^ at 6 weeks, 12 weeks, 24 weeks post-operative were also noted. Data were collected in proforma from hospital records and questionnaires during follow-up. The data was analysed by IBM SPSS Statistics version 16.0.

## RESULTS

**Figure 1:**
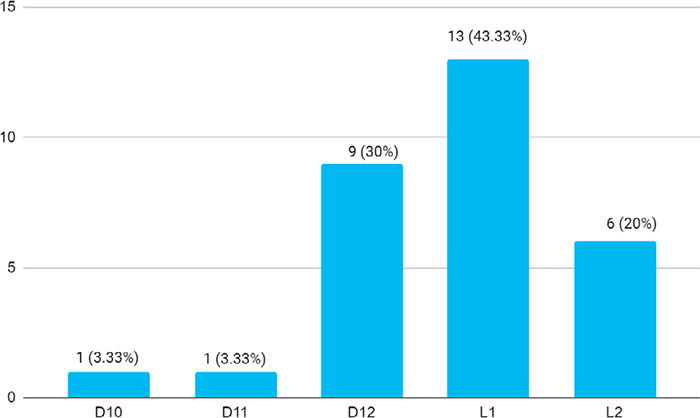
Level of injury (n= 30)

Among 85 patients, the thoracolumbar burst fractures was found in 30 (35.25%) (25.09-45.41, 95% CI). The mean age was 39.73±13.91 years (Range: 16-67 years). A total of 16 (53.33%) were male and 14 (46.66%) were female with a male-female ratio of 1.14. Fall was the mode of injury in 17 (56.66%), road traffic accidents in 9 (30.33%), injury due to impact by heavy objects in 3 (10.33%) and 1 (3.33%) was by animal hit. A total of 17 (56.66%) patients were farmers by occupation. Among the level of injuries, L_1_ injury represented the maximum number of 13 (43.33%) cases (Figure 1).

At presentation, there were 16 (53.33%) cases of ASIA grade E neurological status. Following surgery, 23 (76.66%) cases were of ASIA E neurological status (Table 1).

**Table 1:** Preoperative and postoperative ASIA neurological status (n= 30).

Grade	Pre-op ASIA	Post-op ASIA (6 months)
B	4 (13.33)	4 (13.33)
C	4 (13.33)	-
D	6 (20)	3 (10)
E	16 (53.33)	23 (76.67)

The mean duration of surgery was 117.33±18.43 minutes. The mean interval from hospital admission to surgery time was 2.87±1.97 days. The mean duration of hospital stay was 22.23±23.57 days. The mean kyphotic angle preoperatively was 21.87±6.49° which was 5.93±2.79° during the immediate postoperative period. At the six months, the mean kyphotic angle was 6.57±3.29°. The mean loss of kyphotic angle correction achieved at the six months follow-up was 0.57±1.10° (range 0-4°) (Table 2).

**Table 2:** Kyphotic angle preoperatively and during follow-up visits.

Kyphotic angle	Mean±SD
Pre-operative	21.87±6.49
Immediate post-operative	5.93±2.79
6 montds	6.57±3.29

The mean VAS score at six weeks follow-up was 54.97±17.87 whereas in six months after surgery, it was 14.37±13.98. The mean ODI score at the 6 weeks was 69.10±12.08 which reached the mean of 21.53±15.8 at 6 months follow-up (Table 3).

**Table 3:** VAS score and ODI scores during follow-up visits.

VAS (mm)	Mean±SD
6 weeks	54.97±17.87
12 weeks	32.6±13.97
6 months	14.37±12.07
**ODI**	
6 weeks	65.03±12.13
12 weeks	49.2±13.86
6 months	21.53±15.79

A total of 11 (36.67%) patients had severe pain, 15 (50%) patients had moderate pain and 4 (13.33%) patients had mild pain in VAS score initially. At 6 months 27 (90%) patients had mild pain and 3 (10%) patients had moderate pain. At 6 months, 20 (66.67%) patients had a minimal disability, 7 (23.33%) patients had a moderate disability, 2 (6.67%) patients had a severe disability and 1 (3.33%) patient had a crippled disability. There was 1 (3.33%) complication of superficial infection and 1 (3.33%) with decubitus ulcer which was managed accordingly.

## DISCUSSION

The prevalence of thoraco-lumbar burst fracture was found to 35.25% in our study. In a study the incidence of thoracolumbar burst fracture is found to be 2.4% which has increased to 6.9% over the years.^9^ First lumbar vertebrae fracture was the most common level of injury (43.33%) in our study which was comparable to (51.6%).^10^ There was 64.28% with grade one improvement and 7.14% with grade two improvement in neurological ASIA grading in our study. other studies showed 79.55% had grade one and 20.55% had grade two improvement.^11-14^

Thoracolumbar fractures are the commonest fracture in the axial skeleton and among them burst fractures are the most common.^15^ Around 40% of these injuries are associated with a neurological injury which affects daily life.^16^ Burst fractures with neural and mechanical instability are usually treated surgically. Early management is needed for early neural recovery, to maintain mechanical stability and for early mobilisation and rehabilitation.

The prevalence of thoracolumbar burst fractures managed surgically was 35.25% in our study which was similar to other studies (39.50%).^17^ The mean age in our study was 39.73±13.91 years which was similar to another study with a mean age of 36.7±12.56 years.^18^ Other studies also had similar finding similar age groups.^11,14^ The cause of burst fractures varies. Fall was the most common mode of injury in our study. It accounted for 17 (56.69%) cases, comparable to 58% in a study. study,^18^ and 66% in the study.^11^ It was a very high number of cases of fall injury in another study.^14^ In western countries, road traffic accidents account for the most common cause of injury.^10,12^

In our study it was farmers who were injured the most. Most of the patients were manual workers by occupation according to a study.^14^ As agriculture is the main source of income in our country and male often go out to farms and the jungle to work, it might be the main reason for their injury.

Pedicle screw fixation through posterior approaches is the most common procedure done for the treatment of thoracolumbar fracture with better outcomes regarding operative time, blood loss and complications.^19^ Three-column fixation by pedicle screws controls axial, translational and rotational displacement.^20,21^

The mean duration of surgery was 117 minutes in a published study,^11^ which was similar to our study where the mean duration of surgery was 117.33±13.91 minutes. In contrast to our finding, other studies showed the mean duration of surgery to be 200 minutes, 232 minutes and 240 minutes respectively.^13,18,^ ^22^ These data tell us that may be due to improvements in surgical techniques, the discovery of newer implant systems and upgraded levels of anaesthesia, surgery time is decreasing.^3^

The time interval from injury to surgery time was 5.5 days for one of the studies,^18^ and 14-21 days for the other one.^14^ It was 2.87±1.97 days in our study and 1 day in a study.^23^ Studies have shown better outcomes with less interval from injury to surgery.^24,25^

Mean preoperative, postoperative and final kyphotic angles were 21.87±6.49°, 5.93±2.79° and 6.13±3.29° in our study. Other studies have also shown a similar report whichshowed pre and final kyphotic angles to be 26.8±7.89° and 5.5±3.19° respectively.^11^ Another study showed 26.17±6.49° and 11.58±2.46° respectively.^13^ At the final follow-up the same showed the loss of kyphotic angle correction achieved was 2.83±1.89° whereas our study showed a loss of 0.57±1.15°.^13^

The final VAS score in our study was 14.37±12.08 which was similar to the final VAS score of 16.4±8.9 in the study.^14^ The VAS score was lower in other studies.^11,26^ A total of 27 (90%) cases in our study had mild pain and 3 (10%) had moderate pain at the final follow-up. Similarly, a study also showed 90% of the cases with mild pain at the final follow-up.^14^

A study concluded with a mean ODI score of 29.2% at the final follow-up with 40% of cases reporting as low disability, 27% as moderate disability and 33% as a severe disability.^23^ One of the studies similarly concluded with the mean ODI score at the final follow-up with 25.27% with 59.6% of cases reporting as a mild disability.^14^ Our study showed the final mean ODI of 21.53±15.8 with 66% of the cases as minimal disability, 23.33% as moderate disability, 6.67% as severe disability and 3% as a crippled disability.

Our study has certain limitations. The data was collected retrospectively from the records with a small number of patients from the same institution. The follow-up period was less. So, the findings might not be generalisable to other settings.

## CONCLUSIONS

The prevalence of thoracolumbar burst fracture was similar to other studies done in similar settings. Long-segment posterior fixation is an effective method of treating thoracolumbar burst fractures. This method has a good functional, radiological and neurological outcome with fewer complications.

## References

[1] Pasapula C, MacDonald JW (2004). Thoraco-lumbar fractures.. Orthopaedics Trauma..

[2] Alvine GF, Swain JM, Asher MA, Burton DC (2004). Treatment of thoracolumbar burst fractures with variable screw placement or Isola instrumentation and arthrodesis: case series and literature review.. J Spinal Disord Tech..

[3] Liu L, Gan Y, Zhou Q, Wang H, Dai F, Luo F (2015). Improved monosegment pedicle instrumentation for treatment of thoracolumbar incomplete burst fractures.. Biomed Res Int..

[4] Kim BG, Dan JM, Shin DE (2015). Treatment of thoracolumbar fracture.. Asian Spine J..

[5] Wang F, Zhu Y (2013). Treatment of complete fracture-dislocation of thoracolumbar spine.. J Spinal Disord Tech..

[6] El Masry WS, Tsubo M, Katoh S, El Miligui YH, Khan A (1996). Validation of the american spinal injury association (ASIA) motor score and the national acute spinal cord injury study (NASCIS) motor score.. Spine (Phila Pa 1976)..

[7] Boonstra AM, Schiphorst Preuper HR, Reneman MF, Posthumus JB, Stewart RE (2008). Reliability and validity of the visual analogue scale for disability in patients with chronic musculoskeletal pain.. Int J Rehabil Res..

[8] Fairbank JC, Pynsent PB (2000). The oswestry disability index.. Spine (Phila Pa 1976)..

[9] Fernandez-de Thomas RJ, De Jesus O (2022). StatPearls [Internet]..

[10] Altay M, Ozkurt B, Aktekin CN, Ozturk AM, Dogan O, Tabak AY (2007). Treatment of unstable thoracolumbar junction burst fractures with short- or long-segment posterior fixation in magerl type a fractures.. Eur Spine J..

[11] Chokshi JJ, Shah M (2019). Outcomes of including fracture level in short-segment fixation for thoracolumbar fracture dislocation.. Asian Spine J..

[12] Sapkas G, Kateros K, Papadakis SA, Brilakis E, Macheras G, Katonis P (2010). Treatment of unstable thoracolumbar burst fractures by indirect reduction and posterior stabilization: short-segment versus long-segment stabilization.. Open Orthop J..

[13] Mittal S, Ifthekar S, Ahuja K, Sarkar B, Singh G, Rana A (2021). Outcomes of thoracolumbar fracture-dislocation managed by short-segment and long-segment posterior fixation: a single-center retrospective study.. Int J Spine Surg..

[14] Choudhury AAM, Alam MS, Azad AK, Akhter K (2021). Outcome of unstable thoracolumbar fracture following long segment posterior fixation.. J Bangladesh Coll Physicians Surg..

[15] DeWald RL (1984). Burst fractures of the thoracic and lumbar spine.. Clin Orthop Relat Res..

[16] Hsu JM, Joseph T, Ellis AM (2003). Thoracolumbar fracture in blunt trauma patients: guidelines for diagnosis and imaging.. Injury..

[17] Katsuura Y, Osborn JM, Cason GW (2016). The epidemiology of thoracolumbar trauma: A meta-analysis.. J Orthop..

[18] Carl AL, Tromanhauser SG, Roger DJ (1992). Pedicle screw instrumentation for thoracolumbar burst fractures and fracture-dislocations.. Spine (Phila Pa 1976)..

[19] Bronson WH, Vaccaro AR (2018). Is there a role for anterior augmentation in thoracolumbar burst fractures?. Trong: Indian Spine Journal..

[20] Luque ER (1982). The anatomic basis and development of segmental spinal instrumentation.. Spine (Phila Pa 1976)..

[21] McAfee PC, Farey ID, Sutterlin CE, Gurr KR, Warden KE, Cunningham BW (1989). 1989 Volvo Award in basic science. Device-related osteoporosis with spinal instrumentation.. Spine (Phila Pa 1976)..

[22] Chen F, Kang Y, Li H, Lv G, Lu C, Li J (2017). Treatment of Lumbar Split Fracture-Dislocation With Short-Segment or Long-Segment Posterior Fixation and Anterior Fusion.. Clin Spine Surg..

[23] Canbek U, Karapinar L, Imerci A, Akgun U, Kumbaraci M, Incesu M (2014). Posterior fixation of thoracolumbar burst fractures: is it possible to protect one segment in the lumbar region?. Eur J Orthop Surg Traumatol..

[24] Qadir I, Riew KD, Alam SR, Akram R, Waqas M, Aziz A (2020). Timing of surgery in thoracolumbar spine injury: impact on neurological outcome.. Global Spine J..

[25] Qi C, Xia H, Miao D, Wang X, Li Z (2020). The influence of timing of surgery in the outcome of spinal cord injury without radiographic abnormality (SCIWORA).. J Orthop Surg Res..

[26] Yang WE, Ng ZX, Koh KM, Low SW, Lwin S, Choy KS (2012). Percutaneous pedicle screw fixation for thoracolumbar burst fracture: a Singapore experience.. Singapore Med J..

